# Computational Insights into the Structural Dynamics of MDA5 Variants Associated with Aicardi–Goutières Syndrome and Singleton–Merten Syndrome

**DOI:** 10.3390/biom11081251

**Published:** 2021-08-21

**Authors:** Vijayakumar Gosu, Santanu Sasidharan, Prakash Saudagar, Hak-Kyo Lee, Donghyun Shin

**Affiliations:** 1 Department of Animal Biotechnology, Jeonbuk National University, Jeonju 54896, Korea; gosu@jbnu.ac.kr; 2 Department of Biotechnology, National Institute of Technology, Warangal 506004, Telangana, India; santanu.sasidharan@gmail.com (S.S.); ps@nitw.ac.in (P.S.); 3 Department of Agricultural Convergence Technology, Jeonbuk National University, Jeonju 54896, Korea

**Keywords:** Aicardi–Goutières syndrome, Singleton–Merten syndrome, melanoma differentiation-associated protein 5, *IFIH1*, molecular dynamic simulation

## Abstract

Melanoma differentiation-associated protein 5 (MDA5) is a crucial RIG-I-like receptor RNA helicase enzyme encoded by *IFIH1* in humans. Single nucleotide polymorphisms in the *IFIH1* results in fatal genetic disorders such as Aicardi–Goutières syndrome and Singleton–Merten syndrome, and in increased risk of type I diabetes in humans. In this study, we chose four different amino acid substitutions of the MDA5 protein responsible for genetic disorders: MDA5^L372F^, MDA5^A452T^, MDA5^R779H^, and MDA5^R822Q^ and analyzed their structural and functional relationships using molecular dynamic simulations. Our results suggest that the mutated complexes are relatively more stable than the wild-type MDA5. The radius of gyration, interaction energies, and intra-hydrogen bond analysis indicated the stability of mutated complexes over the wild type, especially MDA5^L372F^ and MDA5^R822Q^. The dominant motions exhibited by the wild-type and mutant complexes varied significantly. Moreover, the betweenness centrality of the wild-type and mutant complexes showed shared residues for intra-signal propagation. The observed results indicate that the mutations lead to a gain of function, as reported in previous studies, due to increased interaction energies and stability between RNA and MDA5 in mutated complexes. These findings are expected to deepen our understanding of MDA5 variants and may assist in the development of relevant therapeutics against the disorders.

## 1. Introduction

Pattern recognition receptors (PRR) are essential for the initiation of innate immune responses, which initially discriminate self- and non-self-components within the organism, thereby regulating responses [1,2]. An example is the retinoic acid-inducible gene 1 (RIG-1)-like receptor, which plays a crucial role in viral infection by sensing viral RNA and initiating/regulating antiviral immune responses [3]. RIG-1-like receptor family comprises of three members: RIG-I, melanoma differentiation-associated protein 5 (MDA5), and laboratory of genetics and physiology 2 [4]. Among these, RIG-1 and MDA5 exhibit similar domain organization and transduce downstream signaling through a common adaptor called mitochondrial antiviral signaling protein. Upon binding to viral RNA, further signaling cascades occur, leading to the production of IFN1 through several signaling intermediates, such as IκB kinase epsilon (IKKε) and TANK Binding Kinase 1 (TBK1) [5,6,7].

RIG-I and MDA5 share similar structural homology, including two repeats of the N-terminal caspase activation recruitment domains, a central DEAD-like or DEAH-like (DExD/H) helicase domain (where x can be any amino acid), and a cysteine-rich C-terminal domain (CTD) [6,8]. The caspase activation recruitment domain interacts with mitochondrial antiviral signaling protein and RNA via the helicase domain. These receptors differ from other pattern recognition receptors, such as Toll-like receptors, which are composed of repeated leucine-rich repeats, required to recognize viral components in a particular pattern. In contrast to Toll-like receptors, RIG-1-like receptors detect viral RNA with a central conserved DExD/H helicase domain. Although CTD plays a role in the autoinhibition of RIG-1 and MDA5, the CTD crystal structure suggests that it plays a crucial role in the high binding affinity and selectivity of double-stranded RNA (dsRNA) [9,10]. However, MDA5 CTD exhibits less binding affinity for dsRNA compared with full-length MDA5 [11].

Despite the homology between MDA5 and RIG-1, they differ with regard to sensing viral infections [12]. RIG-1 detects orthomyxovirus infection, whereas MDA5 senses picarnovirus infection. In particular, MDA5 detects RNA length and secondary structure, in contrast to what RIG-1 detects. Moreover, despite the protective antiviral activity of MDA5, this receptor is involved in several autoimmune and autoinflammatory diseases [12]. Recent studies have reported that MDA5 is activated during certain cancer treatments [13,14], which indicates the importance of understanding the MDA5 activation mechanism at the molecular level. Single nucleotide polymorphisms in the genes encoding pattern recognition receptors are reportedly associated with several diseases [15,16]. These polymorphisms in *IFIH1* encoding MDA5 have been linked to several diseases, such as psoriasis, systemic lupus erythematosus, and type 1 diabetes [17,18,19,20]. It is unclear how these mutations are associated with these diseases; however, reports suggest that a single mutation may induce conformational changes required to influence the function of MDA5 irrespective of RNA binding [21]. In addition, *IFIH1* mutations cause rare disorders such as Singleton–Merten syndrome and Aicardi–Goutières syndrome (AGS) [22,23,24]. AGS is a severe neurological disorder that often results in death during childhood. *IFIH1* mutations located in the central helicase domain suggest alterations in the interaction pattern between dsRNA and MDA5, thereby leading to AGS [23,24]. In particular, L372F, A452T, and R779H are involved in the gain of function and association with AGS. In addition, R822Q has been linked to Singleton–Merten syndrome. Several crystal structures have been solved for MDA5-dsRNA bound complexes [8,25,26]. However, research on mutation-induced conformational changes that may influence the functional mechanism of MDA5 is currently lacking.

In this study, we investigated and compared the conformational changes in wild type and mutant MDA5 upon dsRNA binding. It was previously reported that mutations L372F, A452T, R779H and R822Q results in gain of function [22,23,24]. The dsRNA-bound MDA5 (MDA5^WT^) and mutants (MDA5^L372F^, MDA5^A452T^, MDA5^R779H^, and MDA5^R822Q^) were prepared and subjected to molecular dynamics simulations to understand their structural dynamics. Further, trajectory analysis, principal component analysis (PCA), and residue network analysis were performed to examine the global motions and intramolecular signaling flow of the wild type and mutant MDA5 complexes during simulations. The summary of the work is listed below:

I. Analysis of MDA5 sequence conservation, and stability of mutated sequences (MDA5^L372F^, MDA5^A452T^, MDA5^R779H^, and MDA5^R822Q^);
II. Preparation and MD simulation of MDA5^WT^, MDA5^L372F^, MDA5^A452T^, MDA5^R779H^, and MDA5^R822Q^;
III. Trajectory analysis of MDA5 wild-type and mutant complexes: RMSD, RMSF, SASA, Rg, inter H-bonds, intra-H-bonds, interaction energies, PCA and residue network analysis.

## 2. Materials and Methods

### 2.1. Conservation and Mutant Stability Analyses

First, we extracted the crystal structure of MDA5 bound to dsRNA and ADPNP from the protein databank (ID:4GL2). The description of the structure is as follows, organism: homo sapiens, chain: A, sequence length: 699 residues, domains: “Hel1 (starting residue 298)”, “Hel2i (549)”, “Hel2 (698)”, “Pincer (838)”, “C-terminal domain (900)”, Major molecules: MDA5, dsRNA, ADPNP, Zn ion and missing residues: 392–395, 423–430, 474–477, 544–548, 639–668, 695–698, 746–750, 781–783, 890–899, 943–957, 975–978. The domains are given with the starting residue based on the report [8]. The ConSurf server [27,28] was used to identify the evolutionarily conserved amino acids in MDA5 using either protein structure or sequence with default parameters (https://consurf.tau.ac.il/, accessed on 18 March 2021). First, this server searches for homologous sequences using the HMMER algorithm with an E-value cutoff of 0.0001 in the UNIREF-90 protein database. Multiple sequence alignment is then performed using the MAFFT-L-INS-i method. Finally, the server calculates the phylogeny of homologous sequences and provides the conservation score for each amino acid using the Bayesian method, which classifies the scoring scheme as variable (1–4), intermediate (5–6), and conserved (7–9). Here, the protein sequence of MDA5 was analyzed and the conserved residue was mapped on to the structure (4GL2).

To determine the stability of the protein after single amino acid mutations, I-Mutant 2.0 [29] was used (https://folding.biofold.org/i-mutant//pages/dbMut.html, accessed on 1 June 2021). I-Mutant 2.0 is based on the support vector machine algorithm and can predict the stability of a protein correctly in 80% of cases [29]. The server provides free energy change (DDG) values as a regression estimator and signs of stability change wherein mutated structures with DDG values > 0 are considered to have increased stability and structures with DDG <0 have decreased stability. The functional impact of a mutation was analyzed using the Protein Variation Effect Analyzer, which predicts the impact of a mutation on the biological function of a protein [30]. The server filters sequence variants to identify non-synonymous variants that are functionally important. According to the server, the default threshold score is fixed at −2.5 and variants with scores equal to or below the threshold value are considered deleterious. Likewise, variants with scores above −2.5 are considered neutral.

### 2.2. Preparation of dsRNA-Bound MDA5 Wild Type and Mutant Complexes

MDA5-dsRNA complex structures have been previously solved through crystallography [8]. However, the crystal structures exist with several missing residues, particularly in the loop regions. Hence, to build the missing residues and prepare the MDA5-dsRNA bound complex, we used a dsRNA-bound MDA5 crystal structure (4GL2). The missing residues were built using a Swiss modeler (https://swissmodel.expasy.org/, accessed on 28 April 2020), and then the loops were further refined (only built loops) using mod loop refinement [31,32]. The length of the MDA5 structure considered was 707 residues ranging from 307-1013. As our objective was to assess the structural stability and binding capability of dsRNA upon mutation, we removed the ADPNP molecule from the structure and considered the complex as wild-type MDA5 (MDA5^WT^). To construct mutants, we replaced leucine at position 372 with phenylalanine (MDA5^L372F^), alanine at position 452 with threonine (MDA5^A452T^), arginine at position 779 with histidine (MDA5^R779H^), and arginine at position 822 with glutamine (MDA5^R822Q^) using PyMOL with probable rotamers [33]. Finally, stereochemical properties were checked using the ProQ webserver (https://proq.bioinfo.se/ProQ/ProQ.html, accessed on 14 May 2020) [34], and structural geometries were cleaned for any steric clashes using the Discovery Studio visualizer (Dassault Systems, BIOVIA, San Diego, CA, USA) [35].

### 2.3. Molecular Dynamic Simulations

To assess the structural dynamics of all the five systems (MDA5^WT^, MDA5^L372F^, MDA5^A452T^, MDA5^R779H^, and MDA5^R822Q^), we employed all-atom molecular dynamic simulations using Gromacs 5.1.4 [36,37], as reported in our previous study [38]. The wild-type and mutated complexes were first placed in a dodecahedron box with water molecules, and the distance between the protein surface and box wall was set at 1.2 nm. The Amber-ff99SB-ILDN force field was used for both the protein and dsRNA. Energy minimization was performed using the steepest gradient method with a maximum tolerance of 1000 kJ/mol. Subsequently, equilibration with the NVT and NPT ensemble was performed for 100 ps and 500 ps, respectively, with restraints. Temperature (modified Brendenson thermostat) and pressure (Parrinello–Rahman barostat) couplings were used at 300 K and 1.0 bar for the equilibrations. Finally, production simulations were performed for 200 ns without restraints. Electrostatic interactions such as short- and long-range interactions were applied with 1.0 nm. The Particle Mesh Ewald algorithm was used to maintain the long-range interactions and grid spacing of 0.16 nm for FFT with fourth-order cubic interpolation. A 2 fs time step was used for the simulations, and the 2 ps data were saved for the entire trajectory. Finally, trajectory analysis was performed using the GROMACS analysis tools, and plots were generated using Excel.

### 2.4. PCA and Free Energy Landscape (FEL) Analyses

The concerted motions of structures are essential for their biological functions, and to study the motions of the structures in this study, PCA was conducted for backbone atoms using 50 ps coordinates from the last 100 ns of each complex trajectory. Rotational and translational motions were removed accordingly, and the covariance matrix was computed. From the covariance matrix, the eigenvalues and eigenvectors were analyzed using the gmx anaeig tool. The principal components (PC) with the largest motions were then selected and plotted for comparison. The comparison using the PCs provides the main information about the spread of the datapoints in the phase space, which indicates the global motion of the protein during simulation. To study FEL, the gmx sham tool was used to combine the data points of the reaction coordinates of the PCs with the largest motions. FEL plots were drawn using Mathematica (version 12).

### 2.5. Residue Network Analysis

The impact of mutations on the structure of a protein can be understood in detail by using residue interaction networks. Residue networks were constructed using the NAPS webserver (http://bioinf.iiit.ac.in/NAPS/, accessed on 15 March 2021) [39] for the representative structures of both the wild-type and mutant MDA5 complexes with a non-weighted edge and a Cα-Cα pair maximum threshold of 7 Å. From this analysis, the betweenness centrality (C_B_) values, which may be functionally important for signal transduction within the protein, were determined. C_B_ values play a vital role in suggesting residues that are crucial for protein function. The value indicates the centrality of the node in a residue network. It is calculated by the number of shortest paths from between vertices that pass through a node. Basically, it quantifies the number of times a node can act as a bridge along the paths of any two other nodes. Hence, the high C_B_ value of a particular node (residue) may have an impact on the structural–functional relationship. This allows us to understand the importance of residues involved in long range communications in a protein.

## 3. Results

### 3.1. Conservation, Mutant Stability and Functional Analyses

**Figure 1:**
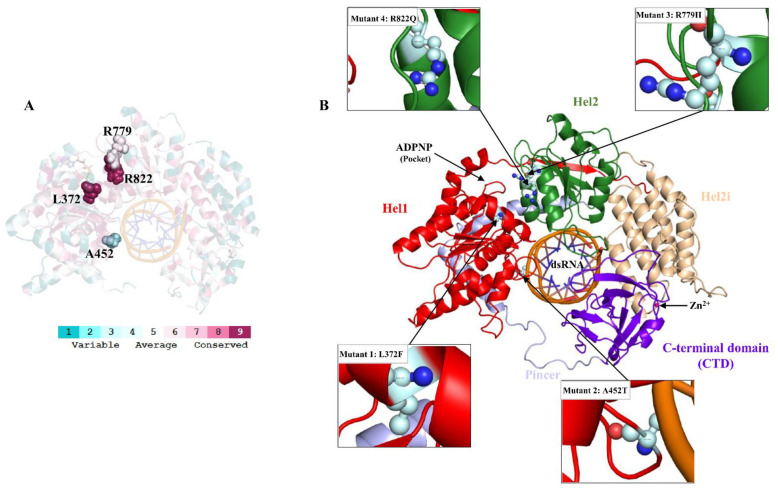
The structure of MDA5. (**A**) The MDA5 structure is shown with evolutionarily conserved residues. The MDA5 sequence/structure was used for conservation analysis. Residues are shown with sphere models according to the scale bar. (**B**) The structure of MDA5 with modelled missing residues. Domains of MDA5 are represented with different colors and labelled. Mutant locations are shown using ball and stick models (cyan color). Zn ion is shown using a sphere model.

The Consurf server analysis suggested that L372 and R822 are largely conserved with a scale of 9, R779 is moderately conserved with a scale of 6, and A452 is variably conserved with a scale of 3 (Figure 1A). These results indicate that L372, R779, and R822 may influence MDA5 function when substituted. Hence, the substitution of L372, A452, R779, and R822 with F, T, H, and Q are associated with AGS and Singleton–Merten syndrome [22,23,24]. We further explored the mutant stability and functional analyses using the I-Mutant 2.0 server. The modelled structures of MDA5^L372F^, MDA5^A452T^, MDA5^R779H^, and MDA5^R822Q^ were submitted to the server and analyzed for DDG values; the values for these variants were −1.13, −0.83, −0.71, and −1.76, respectively. Structures with DDG values < 0 are predicted to be less stable when compared to the wild type, while structures with DDG values > 0 are predicted to be stable. All the structures had decreased stability when compared to the wild type; however, MDA5^A452T^ and MDA5^R779H^ were relatively stable when compared to the other two mutant complexes. Protein Variation Effect analysis of the mutated sequences yielded interesting results, where MDA5^L372F^, MDA5^A452T^, MDA5^R779H^, and MDA5^R822Q^ scored 0.62, −0.825, −2.591 and −3.785, respectively. According to the server, values less than −2.5 are considered deleterious, whereas values greater than or equal to −2.5 are considered non-deleterious. Based on the data, both MDA5^L372F^ and MDA5^A452T^ mutants were considered functionally neutral, while mutant complexes MDA5^R779H^ and MDA5^R822Q^ were predicted to be deleterious.

### 3.2. Structural Dynamics of Wild-Type and Mutant MDA5-dsRNA Bound Complexes

**Figure 2:**
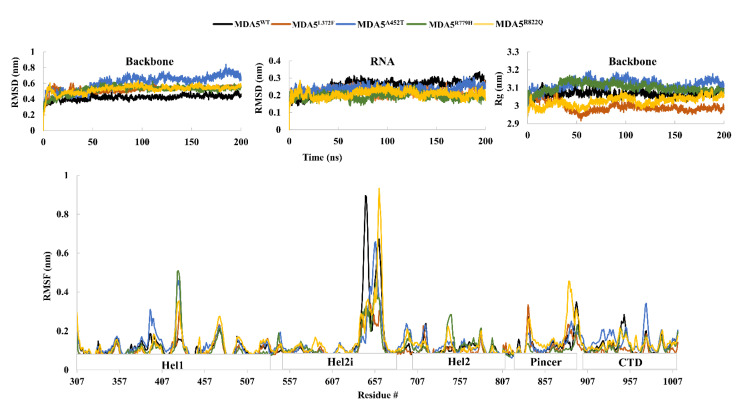
Molecular dynamics trajectory analysis. Protein backbone root mean square deviation (**top left**). Root mean square deviation of dsRNA (**top middle**). Radius of gyration of the backbone atoms of MDA5 (**top right**). Root mean square fluctuations of the backbone atoms of MDA5 (**bottom**).

At first, we prepared the MDA5 wild-type and mutant complexes (Figure 1B) and subsequently subjected to MD simulations. From the MD trajectory of all the complexes, the root mean square deviation (RMSD) of the protein backbone suggested that the wild-type complex (MDA5^WT^) was stable after 50 ns simulations, in contrast to the mutant complexes. In particular, the RMSD of mutant MDA5^A452T^ ranged from 0.5 to 0.8 nm throughout the simulation period, while other complexes showed less variation (Figure 2). The average RMSD values of MDA5^WT^, MDA5^L372F^, MDA5^A452T^, MDA5^R779H^, and MDA5^R822Q^ were 0.4206 nm, 0.5222 nm, 0.6089 nm, 0.5186 nm, and 0.5255 nm, respectively. Moreover, we assessed the RMSD of dsRNA and found it to be highly stable during the simulation. The average RMSD values of the dsRNA complexed with MDA5^WT^, MDA5^L372F^, MDA5^A452T^, MDA5^R779H^, and MDA5^R822Q^ were 0.2527 nm, 0.216 nm, 0.2357 nm, 0.1938 nm, and 0.2078 nm, respectively. The RMSD results indicated that both native and mutant complexes were stable during the simulations (Figure 2).

Further, we assessed the radius of gyration (Rg) for compactness in the folding of the complexes. The complexes MDA5^WT^, MDA5^L372F^, MDA5^A452T^, MDA5^R779H^, and MDA5^R822Q^ had average Rg values of 3.0658 nm, 2.9921 nm, 3.1180 nm, 3.1015 nm, and 3.0246 nm, respectively (Figure 2). Mutant complexes MDA5^A452T^ and MDA5^R779H^ had higher Rg values than that of the wild type, and the remaining two mutant complexes had relatively less compacted folding. These results are consistent with the predictions by I-Mutant 2.0, where MDA5^A452T^ and MDA5^R779H^ were predicted to be stable. Previous reports have suggested that point mutations induce structural alterations that eventually affect the functional properties of a protein [40,41]. To further study the fluctuations with respect to residues in the MDA5 protein, we assessed the root mean square fluctuations (RMSF) for all complexes during the simulations. The largest fluctuations were observed at the C-terminal region of Hel2i in all the complexes. Apart from Hel2i, the region between the wild type and mutants in the central region of Hel1 also exhibited large variations. The other regions, such as the Pincer and C-terminal domains, were stable throughout the simulation. The solvent-accessible surface area (SASA) of the wild-type and mutated complexes was calculated using the gmx sasa GROMACS tool to determine whether there was any reduction in solvent accessibility to different domains (Figure S1). The SASA values for MDA5^WT^, MDA5^L372F^, MDA5^A452T^, MDA5^R779H^, and MDA5^R822Q^ were 385 nm^2^, 377 nm^2^, 384 nm^2^, 383 nm^2^, and 383 nm^2^, respectively. Although other complexes had similar SASA values, MDA5^L372F^ showed both reduced SASA and Rg values. This might be due to the substitution of leucine with phenylalanine, a bulkier group, thereby leading to either a pi-alkyl bond between F372 and L368 or a pi-pi interaction between F372 and F377. In addition, L372 is located adjacent to the ATP binding pocket of MDA5, and substitution at this position might inhibit the hydrolysis of ATP. The analyses also further indicated that there were structural variations among the mutant complexes, in contrast to the wild type.

### 3.3. Hydrogen Bonds and Interaction Energies

**Figure 3:**
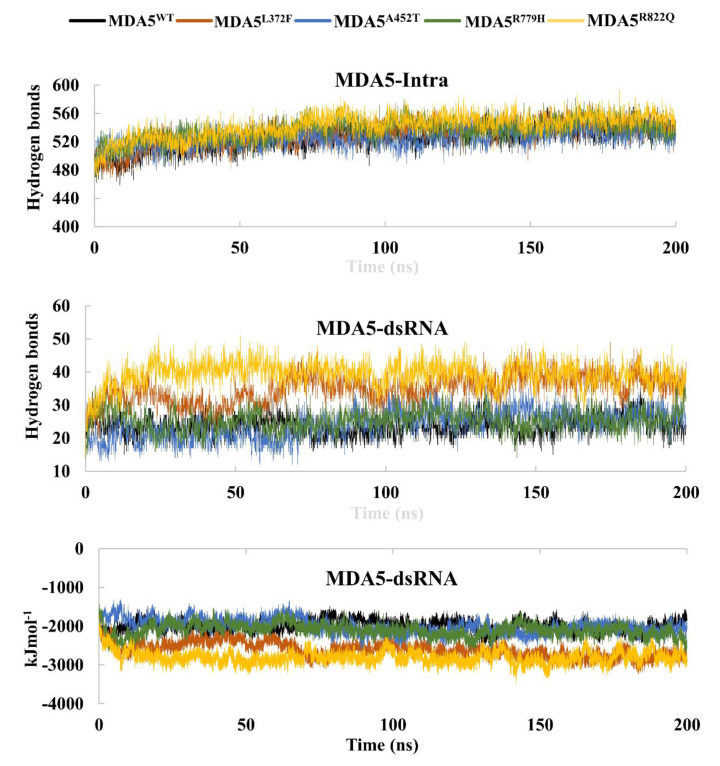
Hydrogen bonds and interaction energies of the molecular dynamics trajectories.

Hydrogen bonds play a crucial role in understanding the binding affinity of RNA towards MDA5. Hence, we used the gmx hbond GROMACS tool to assess the hydrogen bond interactions between dsRNA and MDA5 for all complexes during the simulations. We observed an average of 25 hydrogen bonds in the wild-type complex, whereas in mutant complexes, hydrogen bonds slightly increased during the simulations. In particular, MDA5^L372F^ and MDA5^R822Q^ showed an average of 35 hydrogen bonds between dsRNA and protein (Figure 3). Intra-hydrogen bonds for the proteins were also evaluated to determine whether there were any structural interferences in terms of hydrogen bonds. The wild type had 525 intra H-bonds, whereas the mutated complexes MDA5^L372F^, MDA5^A452T^, MDA5^R779H^, and MDA5^R822Q^ had 529, 524, 536, and 541 hydrogen bonds, respectively. MDA5^R779H^ and MDA5^R822Q^ had more intra-hydrogen bonds than the other mutated complexes. This might be the reason for their stable RMSD, Rg, and SASA values, where the increased intra-hydrogen bonds stabilized the mutated complexes. Further, we performed interaction energy analysis using the re-run option in Gromacs and determined that the wild type exhibited less interaction energy than that exhibited by the mutants (Figure 3). The analyses indicated higher binding affinities between dsRNA and MDA5 variants than that between dsRNA and MDA5 wild-type. Interaction energy was calculated as the sum of the Coloumb-SR and Lennard Jones-SR. For the MDA5 complexes, interaction energies were −1997 kJ/mol, −2612 kJ/mol, −2003 kJ/mol, −2102 kJ/mol, and −2803 kJ/mol for MDA5^WT^, MDA5^L372F^, MDA5^A452T^, MDA5^R779H^, and MDA5^R822Q^, respectively. The overall summary of the trajectories analysis of MDA5^WT^, MDA5^L372F^, MDA5^A452T^, MDA5^R779H^, and MDA5^R822Q^ is given in Table 1. These results suggest that MDA5 variants may induce structural alterations, thereby leading to a gain of function as reported previously.

### 3.4. Principal Component Analysis

**Figure 4:**
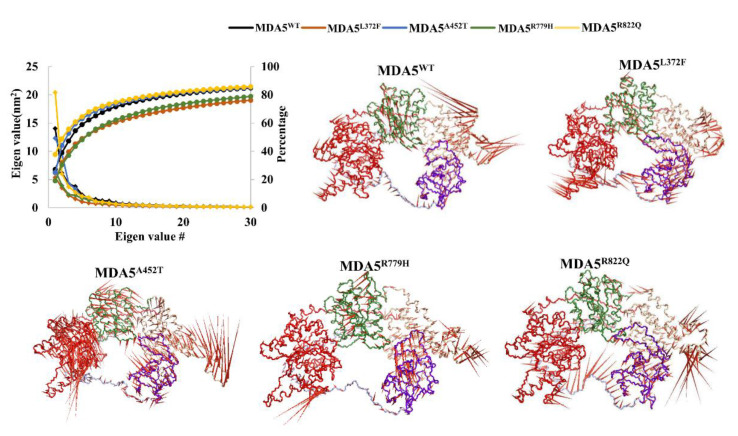
Cumulative percentages and porcupine plots from the principal component analysis. The cumulative percentages of the first 30 principal components are shown (top left corner). Porcupine plots were made using PyMOL for PC1 with 50 frames of the whole trajectory using the filter option. The domains are color coded as per the colors represented in Figure 1B.

**Figure 5:**
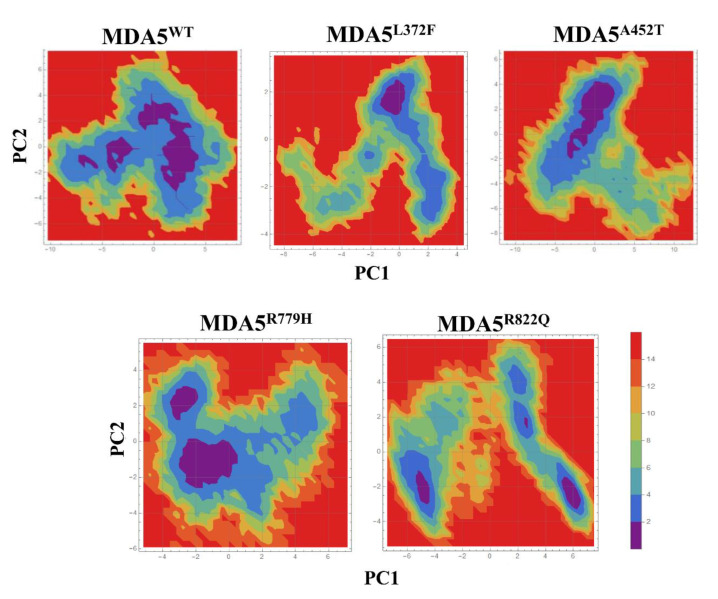
Free energy landscape analysis using PC1 and PC2 coordinates of the molecular dynamics trajectories of all complexes.

To understand the global motions during simulations and the intrinsic dynamics of the complex, we performed PCA on the MDA5 wild type and mutants. The simulation trajectories suggested that the first 30 PCs greatly contributed to the collective motions of the proteins. Moreover, the largest motions were observed in the first three PCs, with cumulative percentages of 60%, 50%, 65%, 45%, and 65% for MDA5^WT^, MDA5^L372F^, MDA5^A452T^, MDA5^R779H^, and MDA5^R822Q^, respectively (Figure 4). The projections for PC1 and PC2, PC2 and PC3, and PC1 and PC3 are shown in Figure S2. The projections for PC1 and PC2 showed that all the complexes were largely spread in the phase with small energy barriers, except for MDA5^R822Q^ and MDA5^A452T^. However, for the PC2 and PC3 projections, a similar spread in the phase space was observed, which strongly indicated less global motion. Again, the projections for PC1 and PC3 revealed a similar spread with a small energy barrier (Figure S2). To understand the local motion in the complexes, we constructed porcupine plots for PC1 using a filter option for all trajectories with 50 frames. We observed that the variations were restricted mainly to the Hel1, Hel2i, and Pincer regions of the mutants, in contrast to the wild type (Figure 4). Furthermore, FEL analysis with PC1 against PC2 strongly suggested the presence of a transition occurring during the simulations with small energy barriers. In particular, MDA5^WT^ showed three clusters in a basin, whereas the mutants did not exhibit large transitions, except for MDA5^R779H^ and MDA5^R822Q^ (Figure 5). These two mutants (MDA5^R779H^ and MDA5^R822Q^) exhibited large transitions, thereby indicating the possibility of large structural alterations in these complexes. These findings suggest that global motions were compromised upon the substitution of the conserved residues.

### 3.5. RNA Interactions with MDA5

We extracted representative structures based on the FEL for each complex. The hydrogen bond interactions between the RNA and protein were analyzed, and we found that the dsRNA interacts with the protein through its phosphate or base group (Table 2). However, the number of contacts was higher between the dsRNA and mutant complexes than between the dsRNA and wild-type MDA5. This strongly suggested the possibility of a higher binding affinity in mutant complexes than in wild-type MDA5.

### 3.6. Residue Network Analysis

**Figure 6:**
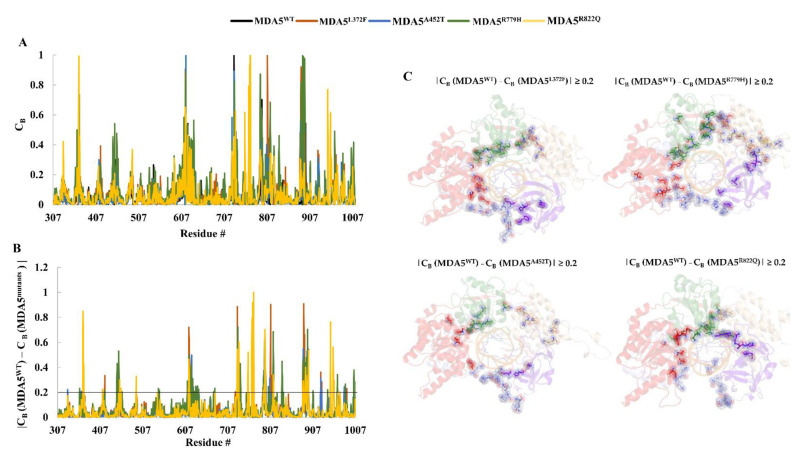
Betweenness centrality (C_B_) analysis. C_B_ values were calculated for each complex using the representative structures. (**A**) The plot represents the C_B_ of each complex. (**B**) The plot represents the difference in C_B_ of MDA5 wild type and mutants based on the condition |C_B_ MDA5^WT^ − C_B_ MDA5^mutants^| ≥ 0.2 (absolute values). (**C**) The selected residues are mapped on the MDA5 and its variant structures. The residues are color coded as per the colors represented in Figure 1B.

We performed residue network analysis, and the results showed crucial structural rearrangements in mutants in contrast to the wild-type protein. We used the NAPS webserver and constructed a residue-to-residue network with a cut-off distance of 0.7 nm. The C_B_ (betweenness centrality) was also computed, as it is important to understand intramolecular signaling flow. The study used these conditions, which showed the variation between the wild type and mutant, thereby presenting insights into the variations in mutants. A high C_B_ value node is typically associated with the function of the complex. Considering this, we calculated the C_B_ for all the complexes (Figure 6), further, we analyzed the C_B_ difference (C_Bd_) between MDA5 wild-type and mutants, using the condition |C_B_ (MDA5^WT^) − C_B_ (MDA5^mutants^)| ≥ 0.2 and mapped the respective residues on the structure to understand the difference between the wild type and mutated complexes. We observed the similar distribution of crucial residues among the mutant complexes and they shared approximately 50% of the residues. This result suggests that the intra residue signaling varies in the MDA5 mutant complexes compared to the MDA5 wild-type, thereby influencing the functional mechanism. (Figure 6 and Table 3). Notably, the residues around the central region of the MDA5 accommodating the dsRNA may play a crucial role in conformational changes in the mutants. However, these residues require further investigation. The variations in C_B_ indicate how point mutations can induce allosteric changes in the protein.

## 4. Discussion

AGS and Singleton–Merten syndrome are two genetic disorders characterized by several physical defects. These disorders are caused by a single nucleotide polymorphism in the *IFIH1* , which codes for MDA5. MDA5 is a crucial PRR that is involved in innate immunity by recognizing viral RNA. The variants of MDA5 are associated with several diseases in human. In particular, L372F, A452T, R779H and R822Q have been associated with AGS and Singleton–Merten syndrome and recent studies have suggested that these variants result in gain of function, thereby leading to the disease [22,23,24]. A recent report on cryo-EM structures solved for MDA5 filaments showed that mutations of the filament-forming residues can alter the function [25]. In addition, the chicken MDA5 dimer structure (2MDA5 and one dsRNA) suggested Head-Head configuration upon binding to short/long dsRNA, thereby forming a filament [26]. Several computational studies have previously reported the use of molecular dynamics’ simulations to understand the dynamic nature as well as the structure–function relationship of the proteins and ligand bound complexes [38,40,41,42,43,44]. Hence, in order to understand the reason behind the gain of function associated with MDA5 variants, we performed long-range simulations to identify the structural changes that may influence the functional properties of MDA5 in this study. Five different systems were selected for the study: MDA5^WT^, MDA5^L372F^, MDA5^A452T^, MDA5^R779H^, and MDA5^R822Q^.

We mainly used traditional analyses such as RMSD, Rg, RMSF, and hydrogen bond analysis, which altogether suggest that structural variations occur upon mutation in MDA5. From stability and functional analyses using web servers, it was predicted that MDA5^L372F^, MDA5^A452T^, MDA5^R779H^, and MDA5^R822Q^ were relatively less stable than the wild type, and MDA5^R779H^ and MDA5^R822Q^ were predicted to be deleterious. Although the RMSD values of the backbone of the complexes were higher than that of the wild type, the RMSDs of dsRNA chains were stable throughout the simulation. Rg analysis showed the structural compactness of the complexes, especially MDA5^L372F^. The MDA5^L372F^ SASA values were also reduced, corroborating the Rg analysis, which might be due to the pi-alkyl or pi-pi interaction of the substituted phenylalanine residue. In addition, MDA5^R822Q^ displayed increased stability in terms of RMSD, Rg, SASA, and intra-hydrogen bonds compared to the other complexes. Therefore, we hypothesize that residue substitution might provide adequate stability for the MDA5 protein to gain function. The MDA5^A452T^ complex had the highest relative instability with respect to the RMSD of the backbone, increased Rg, and increased SASA values. Interestingly, when the interaction energies were analyzed, the mutated complexes had higher interaction energies than that of MDA5^WT^ in the following order: MDA5^R822Q^ > MDA5^L372F^ > MDA5^R779H^ > MDA5^A452T^. These results corroborated another trajectory analysis showing that the MDA5 mutated complexes were more stable than the wild type. Further, the variation in the dominant motions of MDA5 wild-type and mutant complexes suggest that large structural modifications occur to alter the intrinsic dynamics within the complexes (Figure 4) In addition, the betweenness centrality (C_B_) indicates the distribution of crucial residues in the complexes; in particular, C_B_ differences between MDA5 wild-type and mutants suggest the similar distribution of functionally important residues within the complexes (Figure 6 and Table 3). Observed shared residues (~50%) among the mutant complexes in crucial domains of MDA5 indicate that these domains may undergo conformational changes, and also suggest the impact on the stability between dsRNA and MDA5. As a result, these mutations may lead to the syndromes through gain of function. MDA5 forms a long filament complex upon binding to dsRNA and subsequently undergoes RNA-dependent ATP hydrolyzation; hence, it is necessary to perform microsecond simulations along with the ATP-bound state. Although our study is limited to 200 ns simulations, our findings may help deepen the understanding of the mechanistic properties of wild-type and mutant MDA5 upon binding to dsRNA, as well as provide information for basic research on the impact of MDA5 variants that are involved in several diseases This study also provides insights into the general thought that point mutations induce conformational changes that might regulate the function of a protein.

## 5. Conclusions

MDA5 is an important PRR coded by *IFIH1*, and the variants of MDA5 studied in this work has been previously reported to result in gain of function. The gain of function from the MDA5 variants leads to diseases such as Singleton–Merten syndrome and Aicardi–Goutières syndrome in humans. Therapeutics for these syndromes may be defined and designed if the molecular properties and dynamic nature of dsRNA-bound MDA5 mutant complexes are well understood. Therefore, to decipher the conformational changes that occur upon mutations of MDA5, we simulated and analyzed the trajectories of wild-type and four mutated dsRNA-bound MDA5 complexes. The all-atom MD simulation of MDA5 wild-type and mutated complexes (MDA5^L372F^, MDA5^A452T^, MDA5^R779H^, and MDA5^R822Q^) revealed increased stability of the mutated complexes when compared to MDA5 wild-type. The hydrogen bond interactions between MDA5 and the dsRNA were also higher in the mutant complexes than the MDA5^WT^. The varied global motions in wild-type and mutant complexes suggest that point mutations induced certain conformational changes. In addition, the betweenness centrality of the residue networks suggested the crucial residues that might be responsible for the functional variation in the MDA5 mutant complexes compared to wild-type. From these results, we assume that MDA5 variants induce specific structural changes in order to increase the dsRNA-MDA5 binding affinity, which may lead to further signaling and associated diseases.

## Figures and Tables

**Table 1:** Summary of trajectory analysis results of MDA5^WT^, MDA5^L372F^, MDA5^A452T^, MDA5^R779H^, and MDA5^R822Q^ simulations. Standard deviations are given in the brackets.

Trajectory Analysis	MDA5^WT^	MDA5^L372F^	MDA5^A452T^	MDA5^R779H^	MDA5^R822Q^
RMSD (nm)	0.4206 (0.03)	0.5222 (0.04)	0.6089 (0.09)	0.5186 (0.05)	0.5255 (0.04)
RMSD(dsRNA) (nm)	0.2527 (0.03)	0.216 (0.02)	0.2357 (0.02)	0.1938 (0.02)	0.2078 (0.02)
Rg (nm)	3.0658 (0.01)	2.9921 (0.02)	3.1180 (0.02)	3.1015 (0.02)	3.0246 (0.02)
SASA (nm^2^)	385 (7)	377 (10)	384 (9)	383 (7)	383 (7)
Intra H-bonds	525 (16)	529 (17)	524 (13)	536 (14)	541 (27)
Interaction energies (kJ/mol)	−1997 (143)	−2612 (194)	−2003 (186)	−2102 (165)	−2803 (162)

**Table 2:** Hydrogen bond interactions between dsRNA and MDA5.

Domains	MDA5^WT^	MDA5^L372F^	MDA5^A452T^	MDA5^R779H^	MDA5^R822Q^
Hel1	K365	K365	K365	K365	N364
	K397	V366	H447	T394	V366
	Q415	G392	N449	Q395	G392
	K450	T394		H447	T413
	A452	Q395		N449	Q415
		T413			
		Q415			
		H447			
		N449			
Hel2i	Q576	Q576	Q576	Q580	Q576
	Q580	E579	Q580	Q584	Q580
	Q584	Q580	Q584		K587
	K587	K587	K587		
		R605			
Hel2	K726	K726	K726	K726	K726
	R728	R728	R728	R728	R728
	G756	A757	G756	G756	A757
	A757	H759	A757	A757	S761
	Q771	S760	Q768	V791	K764
	V791	S760	Q771		Q768
		Q771	I814		Q771
Pincer	K889	K889	K885	K889	K885
	R890	R890	K889	N891	T888
		A893		K894	R890
C-terminal domain	M926	E924	K949	D953	E924
	H927	L947	R985	Y954	M926
	N944	D953	K1001	K975	K949
	K949	K975		K983	D953
		K983		K1001	I956
		R985		K1002	V973
		K1001			K975
		K1002			K983
					R985
					K1001
					K1002

**Table 3:** The table represents the residue numbers from MDA5 mutants for which the C_Bd_ between MDA5 wild-type and mutants is 0.2 or more. Bold type distinguishes the residues common for two or more mutants.

**|C_B_ (MDA5^WT^) − C_B_ (MDA5^L372F^)| ≥ 0.2**
418, 447, 449, **451**, **453**, **492**, **616**, **617**, **618**, **621**, **622**, **625**, 725, **730**, **733**, 790, **791**, **792**, **793**, **794**, **795**, 808, 810, **812**, **814**, 884, 886, **887**, **888**, **890**, **894**, **896**, **897**, **926**, **928**, 987, 1010
**|C_B_ (MDA5^WT^) − C_B_ (MDA5^A452T^)| ≥ 0.2**
331, 363, 368, **451**, 615, **621**, **622**, **625**, 626, 634, **636**, **733**, **791**, **792**, **793**, **794**, **795**, 806, **812**, **885**, **887**, **888**, **889**, 891, **892**, **894**, **896**, **897**, **926**, **928**, 981
**|C_B_ (MDA5^WT^) − C_B_ (MDA5^R779H^)| ≥ 0.2**
359, 418, 447, 449, **451**, 455, 459, **492**, 544, 546, 612, **616**, **618**, **621**, **625**, 628, 632, **636**, 639, 674, 677, **730**, 731, **733**, 734, 735, 737, 790, **791**, **792**, **794**, **795**, 796, 803, **812**, **814**, **815**, 835, 836, **885**, **887**, **888**, **889**, **890**, **892**, 893, **895**, **897**, 908, 942, 965, 984, 1004, 1009
**|C_B_ (MDA5^WT^) − C_B_ (MDA5^R822Q^)| ≥ 0.2**
367, 368, 369, 413, **453**, 456, **492**, 615, **616**, **617**, **730**, 731, **733**, 755, 764, 766, 768, **791**, **793**, **795**, **814**, **815**, 884, **885**, **890**, **892**, **894**, **895**, **896**, **897**, 949, 955, 956, 957, 971

## Data Availability

The raw data supporting the conclusions of this study are available on request from the corresponding author. The data are not publicly available due to larger in size.

## Supplementary Materials

The following are available online at https://www.mdpi.com/article/10.3390/biom11081251/s1, Figure S1: Solvent accessible surface area, Figure S2: Projection of principal components on to the phase space.
