# Supplementary material for: Computational Insights into the Structural Dynamics of MDA5 Variants Associated with Aicardi–Goutières Syndrome and Singleton–Merten Syndrome
Source: Biomolecules. 2021 Aug 21;11(8):1251. doi: 10.3390/biom11081251 (PMC8393256; doi:10.3390/biom11081251)
Supplement: Supplementary file 1 [file biomolecules-11-01251-s001.zip › biomolecules-1320076-supplementary.pdf]

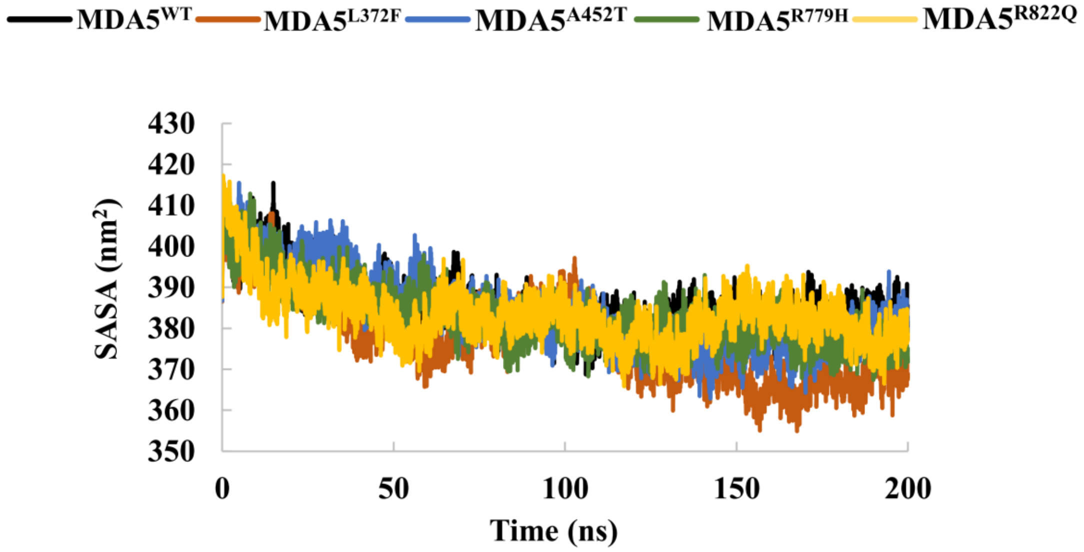

Figure S1: Solvent accessible surface area

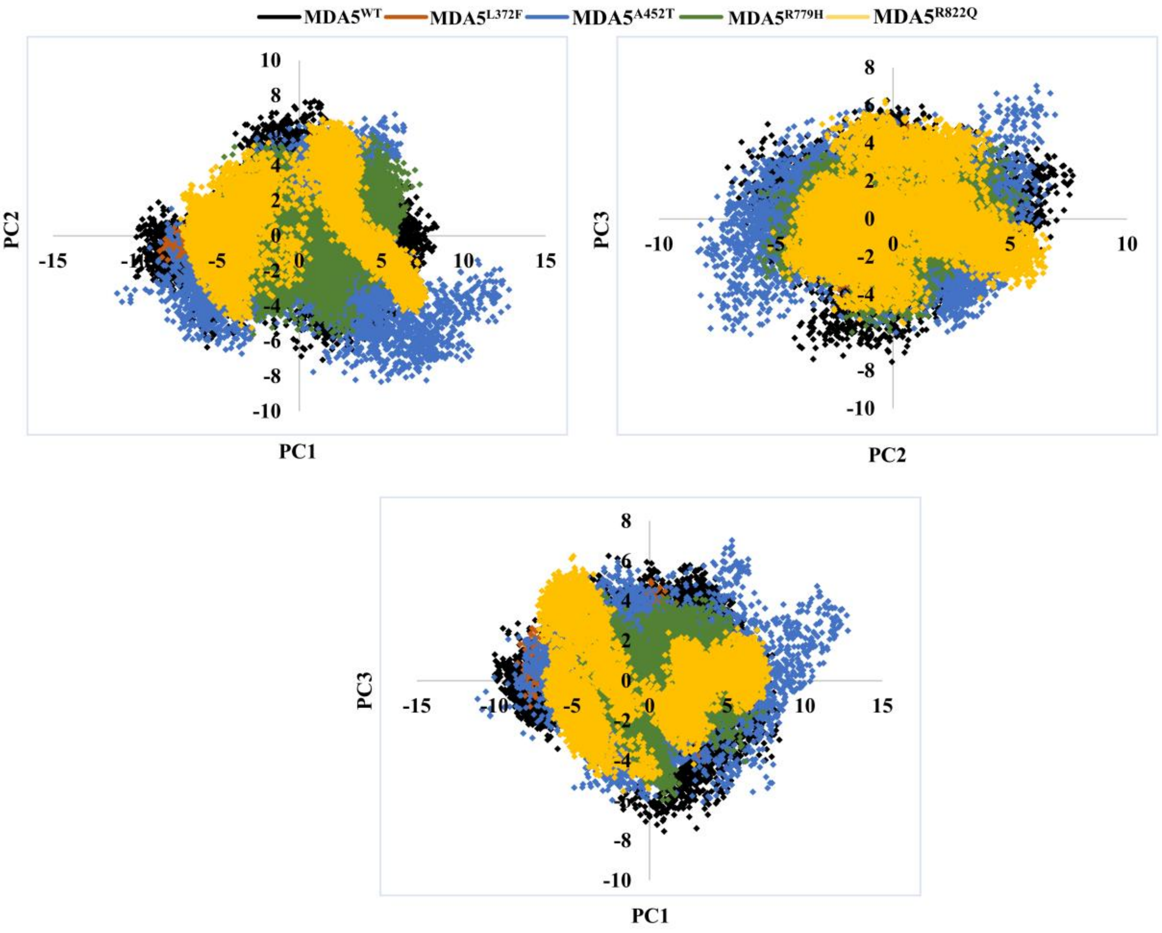

Figure S2: Projection of principal components on to the phase space.
